# Supplementary material for: Whole genome assemblies of Zophobas morio and Tenebrio molitor
Source: G3 (Bethesda). 2023 Apr 1;13(6):jkad079. doi: 10.1093/g3journal/jkad079 (PMC10234394; doi:10.1093/g3journal/jkad079)
Supplement: jkad079_Supplementary_Data [file jkad079_supplementary_data.zip › Kaur_et_al_2023_File_S1.pdf]

Supplementary Materials for

Kaur S, Stinson SA, diCenzo GC. 2023. Whole genome assemblies of *Zophobas morio* and *Tenebrio molitor*. G3: Genes | Genomes | Genetics.

George diCenzo
Email: george.dicenzo@queensu.ca

This PDF file includes:

Figures S1 to S5
Legends for Tables S1-S3

Other supplementary materials for this manuscript include the following:

Tables S1-S3

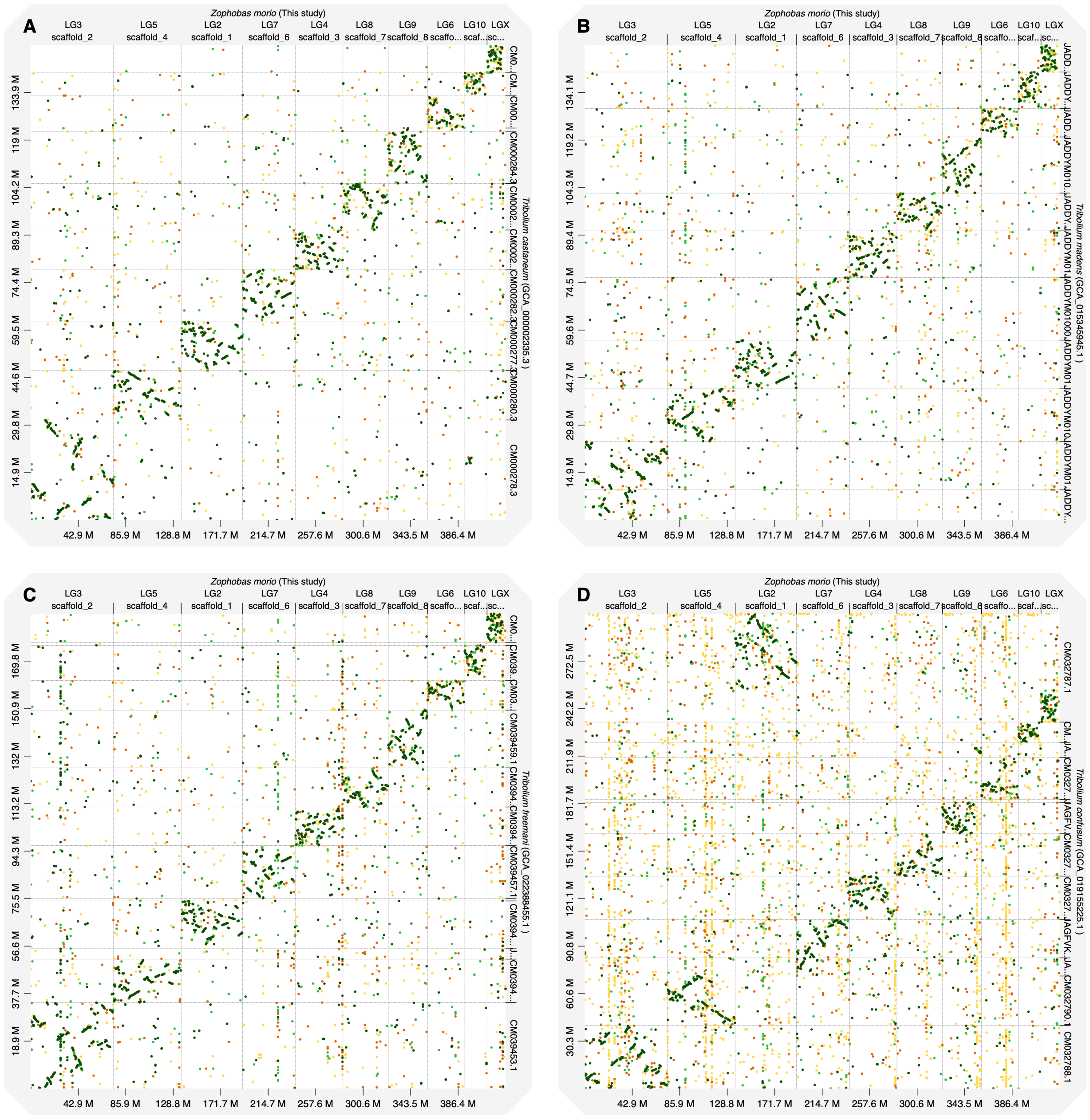

Figure S1. Macrosynteny between *Zophobas morio* and *Tribolium* spp.. Dot plots are shown comparing the genomes of *Z. morio* (this study) and (**A**) *T. castaneum* (GCA_000002335.3), (**B**) *T. madens* (GCA_015345945.1), (**C**) *T. freemani* (GCA_022388455.1), and (**D**) *T. confusum* (GCA_019155225.1). Dot plots were created using D-Genies with the minimap2 aligner. Prior to the dot plot analyses, genomes were filtered to remove scaffolds less than 1 Mb in length for visualization purposes. Dashed grey lines delineate scaffolds. The dot colours indicate the average percent identity of the match. Linkage groups have been added to the *Z. morio* scaffolds according to a previous *Z. morio* genome assembly available through the NCBI Assembly database (GCA_022388465.1).

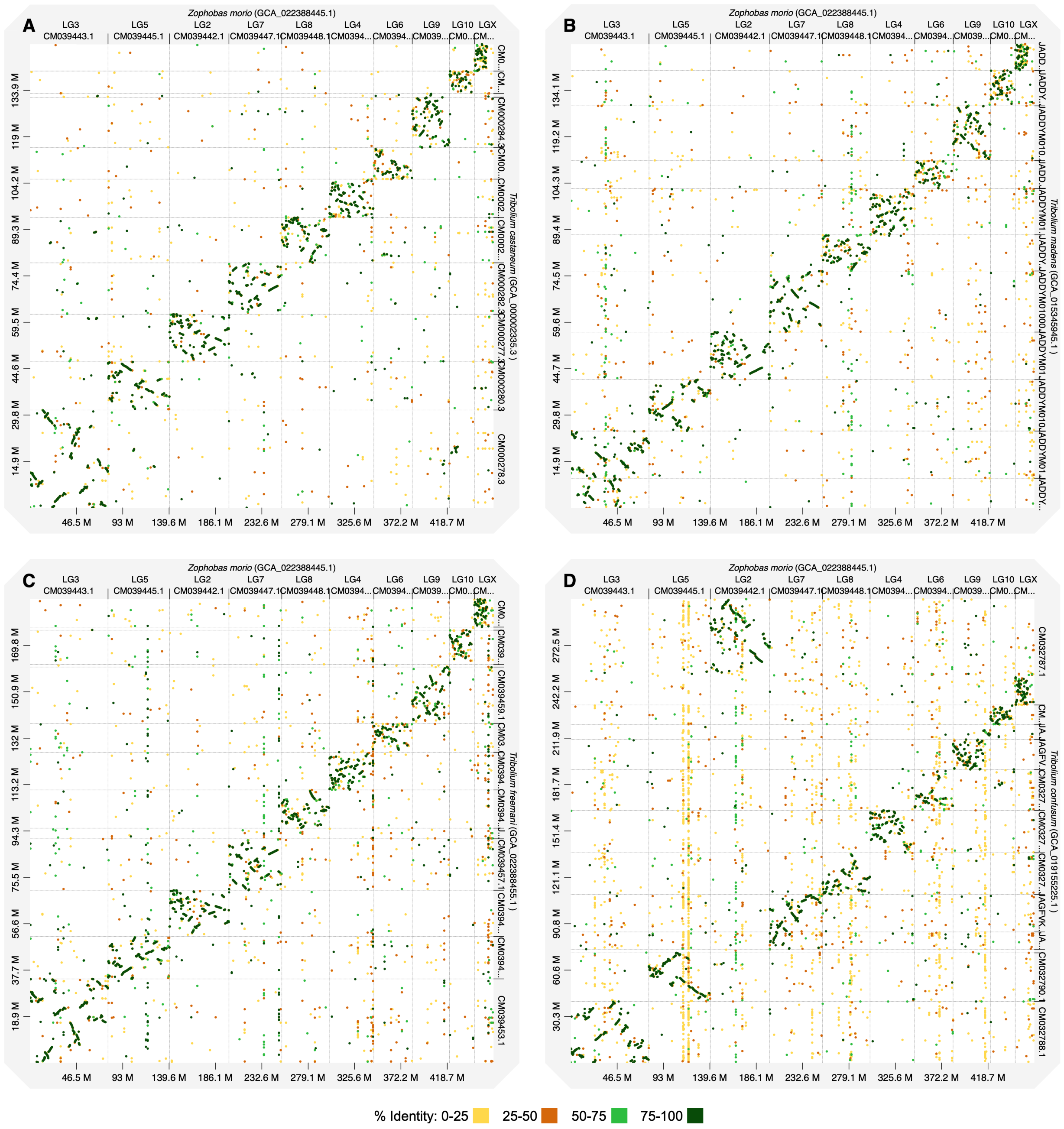

Figure S2. Macrosynteny between *Zophobas morio* and *Tribolium* spp.. Dot plots are shown comparing the genomes of *Z. morio* (GCA_022388445.1) and (**A**) *T. castaneum* (GCA_000002335.3), (**B**) *T. madens* (GCA_015345945.1), (**C**) *T. freemani* (GCA_022388455.1), and (**D**) *T. confusum* (GCA_019155225.1). Dot plots were created using D-Genies with the minimap2 aligner. Prior to the dot plot analyses, genomes were filtered to remove scaffolds less than 1 Mb in length for visualization purposes. Dashed grey lines delineate scaffolds. The dot colours indicate the average percent identity of the match. Linkage groups have been added to the *Z. morio* scaffolds as described in the NCBI Assembly database (GCA_022388465.1).

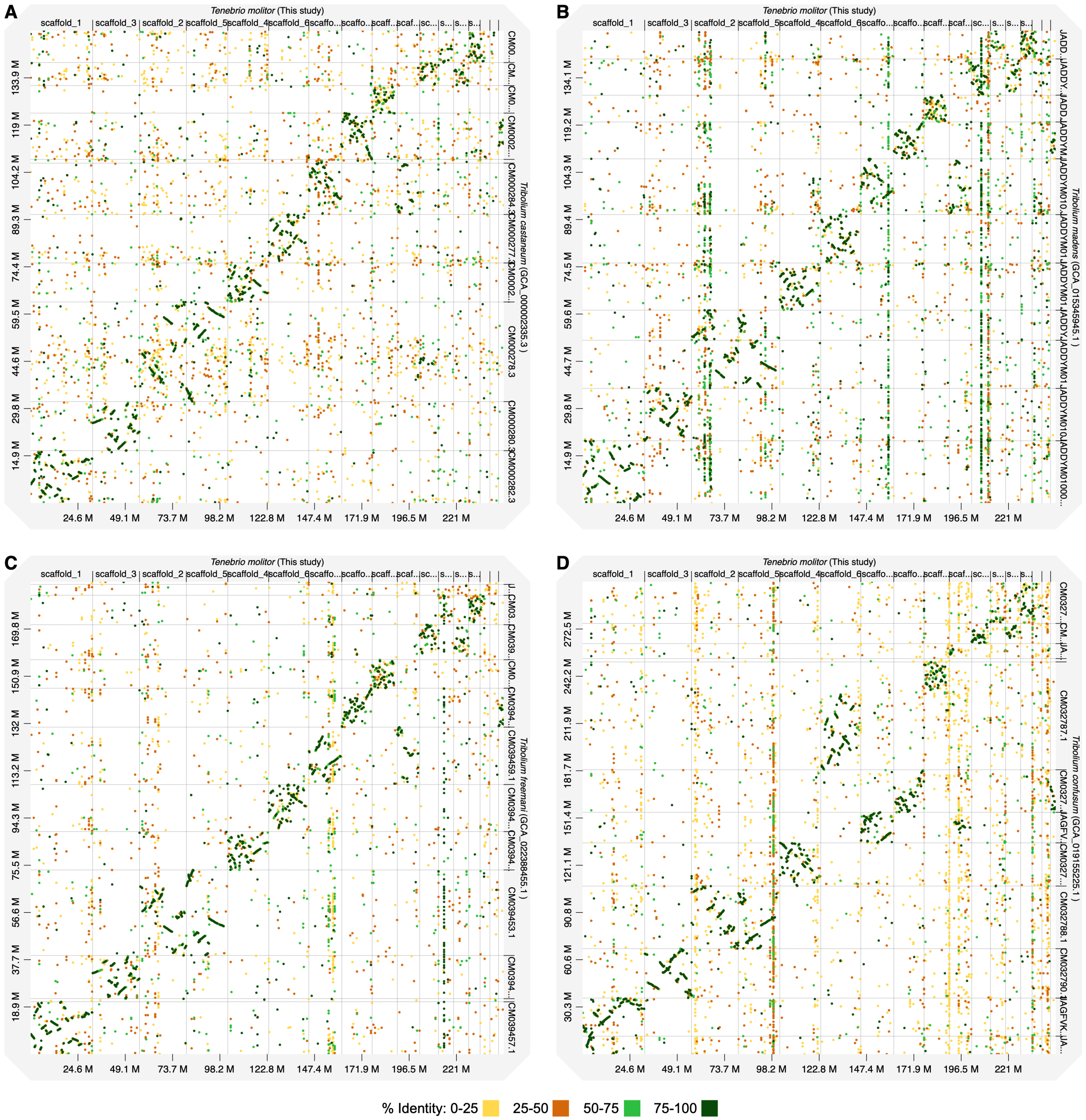

Figure S3. Macrosynteny between *Tenebrio molitor* and *Tribolium* spp.. Dot plots are shown comparing the genomes of *T. molitor* (this study) and (**A**) *T. castaneum* (GCA_000002335.3), (**B**) *T. madens* (GCA_015345945.1), (**C**) *T. freemani* (GCA_022388455.1), and (**D**) *T. confusum* (GCA_019155225.1). Dot plots were created using D-Genies with the minimap2 aligner. Prior to the dot plot analyses, genomes were filtered to remove scaffolds less than 1 Mb in length for visualization purposes. Dashed grey lines delineate scaffolds. The dot colours indicate the average percent identity of the match.

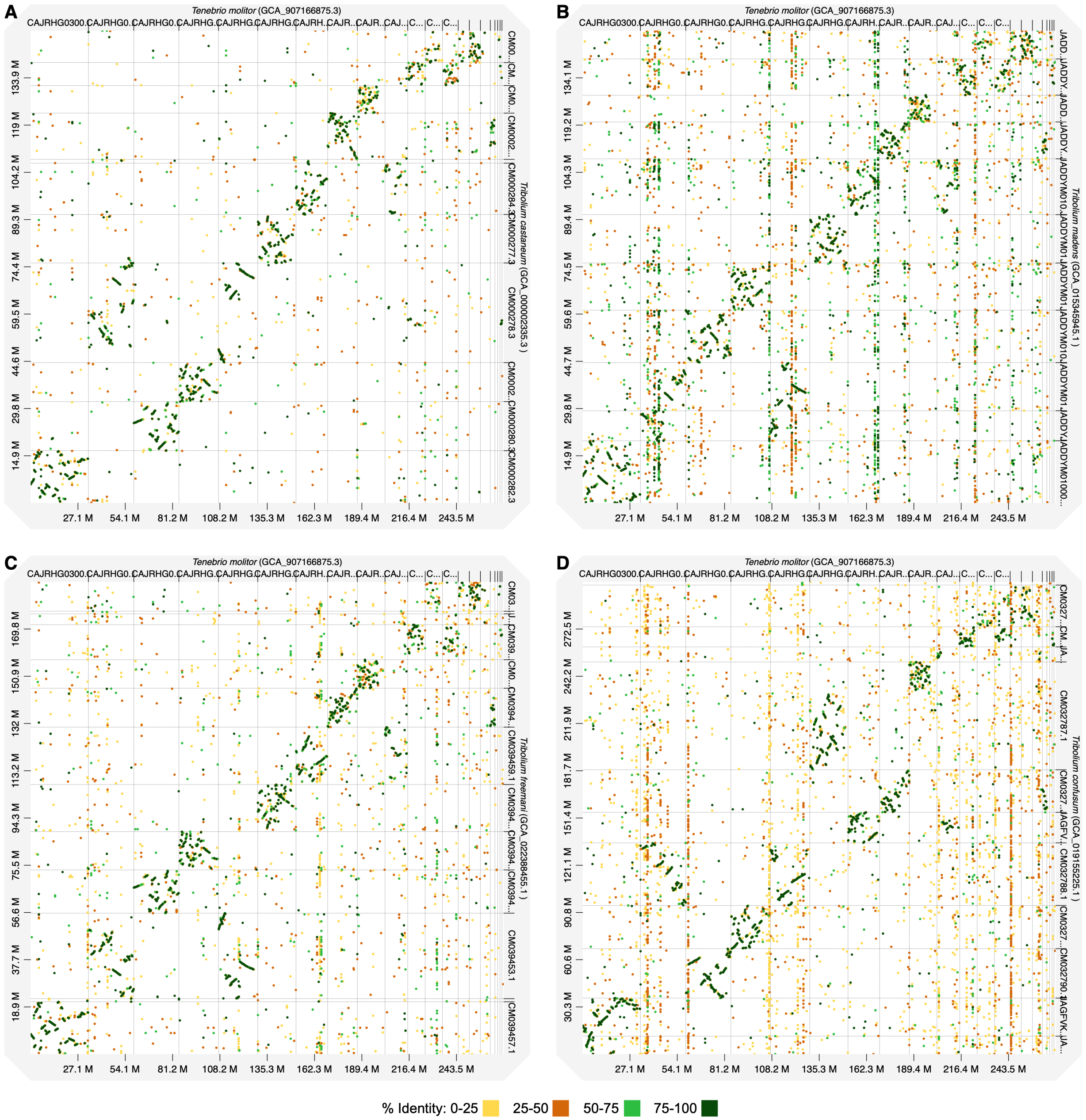

Figure S4. Macrosynteny between *Tenebrio molitor* and *Tribolium* spp.. Dot plots are shown comparing the genomes of *T. molitor* (GCA_907166875.3) and (**A**) *T. castaneum* (GCA_000002335.3), (**B**) *T. madens* (GCA_015345945.1), (**C**) *T. freemani* (GCA_022388455.1), and (**D**) *T. confusum* (GCA_019155225.1). Dot plots were created using D-Genies with the minimap2 aligner. Prior to the dot plot analyses, genomes were filtered to remove scaffolds less than 1 Mb in length for visualization purposes. Dashed grey lines delineate scaffolds. The dot colours indicate the average percent identity of the match.

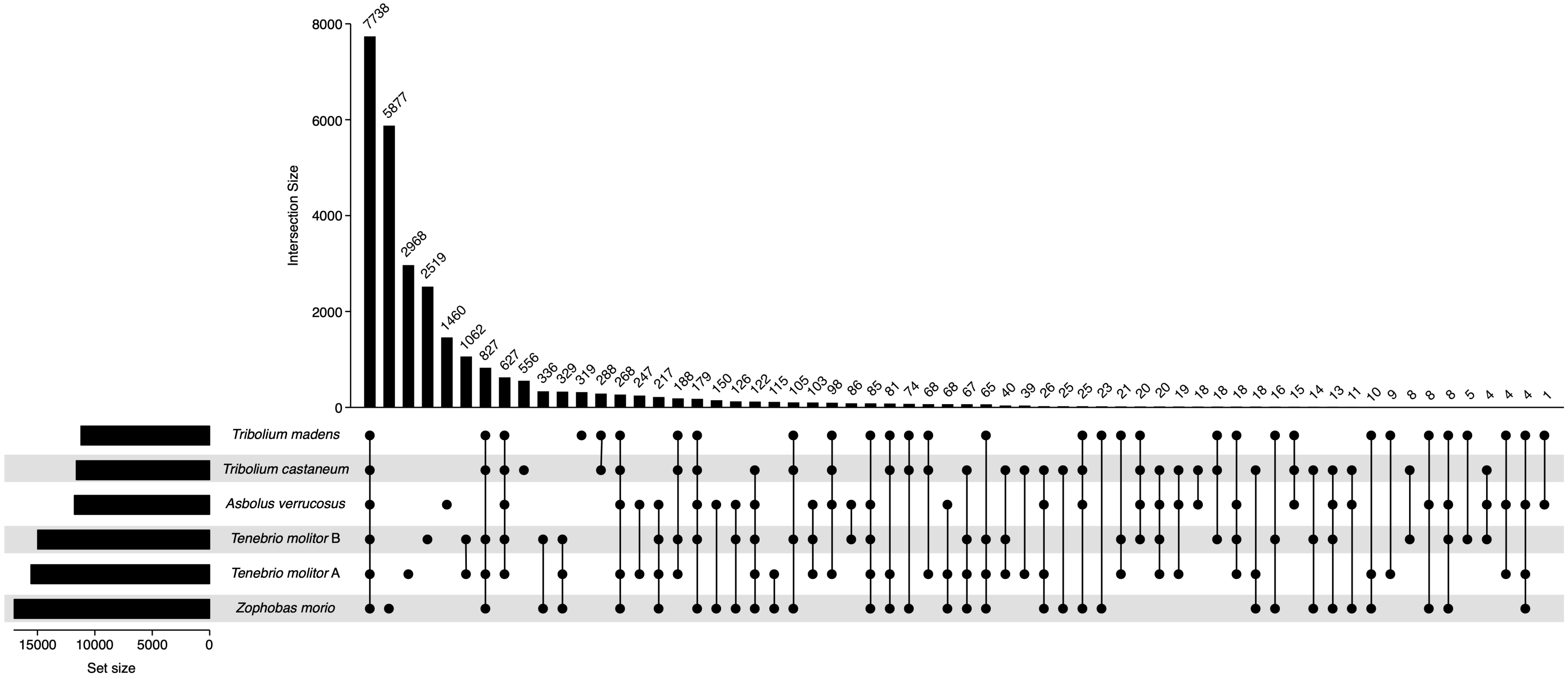

Figure S5. Conservation of gene families across the family Tenebrionidae. Orthofinder was used to group the annotated proteins of *A. verrucosus* (GenBank accession GCA_004193795.1), *T. castaneum* (RefSeq accession GCF_000002335.3), *T. madens* (RefSeq accession GCF_015345945.1), *T. molitor* A (GenBank accession GCA_907166875.3), *T. molitor* B (this study), and *Z. morio* (this study) into gene families. Gene family conservation was summarized using UpSetR. The set size shows the total number of gene families in a given proteome, while the intersect size shows the number of gene families conserved across the indicated proteomes.

LEGENDS FOR SUPPLEMENTARY TABLES

Table S1. Assembly statistics, including BUSCO scores, for 20 *Zophobas morio* and 20 *Tenebrio molitor* genome assemblies are provided. The assembly selected to move forward to annotation is indicated in boldface font.

Table S2. Eukaryota BUSCO scores are reported for the new and previously published *Zophobas morio* and *Tenebrio molitor* genome assemblies and proteomes. Scores were calculated using the BUSCO eukaryota_odb10 dataset. No values are reported for the proteome of the previously published *Z. morio* genome as gene predictions were not performed for this genome.

Table S3. Classification of the LINEs and DNA transposons identified in the *Zophobas morio* and *Tenebrio molitor* genome assemblies.
